# Effect of Denosumab on Bone Health, Vascular Calcification, and Health-Related Quality of Life in Hemodialysis Patients with Osteoporosis: A Prospective Observational Study

**DOI:** 10.3390/jcm13051462

**Published:** 2024-03-02

**Authors:** Hyunsook Kim, Eun Ju Lee, Siyun Woo, Sohee Rho, Ji Yong Jung

**Affiliations:** 1 Department of Health Sciences and Technology, Gachon University, Incheon 21565, Republic of Korea; khss@gachon.ac.kr; 2 Division of Nephrology, Department of Internal Medicine, Gachon University Gil Medical Center, Incheon 21565, Republic of Korea; woohax5@gilhospital.com (E.J.L.); woo-ga@gilhospital.com (S.W.);

**Keywords:** denosumab, CKD-MBD, bone mineral density, hemodialysis

## Abstract

**Background**: Osteoporosis is common in hemodialysis (HD) patients, contributing to cardiovascular risks. Limited research exists on denosumab’s efficacy in this group. Our study explores denosumab’s effects on bone turnover markers (BTMs) and vascular calcification in chronic kidney disease–mineral bone disorder (CKD-MBD) patients. **Methods**: In a prospective single-center study, we investigated the effects of denosumab over 2 years on 30 HD patients from a cohort of 185. Annual assessments of bone mineral density (BMD), vascular calcification, and health-related quality of life (HRQL) were conducted and compared with an untreated group. Mineral and bone parameters were analyzed at specific intervals in the treatment group. **Results**: Denosumab notably raised femoral BMD in the initial year. Most bone turnover markers (BTMs) decreased, except for osteocalcin. Changes in T50 correlated with BTMs. Pre-denosumab supplementation of calcium and vitamin D helped manage mineral imbalances. Post denosumab, parathyroid hormone (PTH) levels increased initially, stabilizing after 3 months. No significant changes occurred in vascular calcification or HRQL. **Conclusions**: Denosumab exhibited varying effects on BMD improvement, with a stronger impact in the first year that diminished in the second year. Early PTH monitoring was crucial, and extending the administrative period may enhance BMD outcomes compared to the general population.

## 1. Introduction

Chronic kidney disease–mineral and bone disorder (CKD-MBD) is common in hemodialysis (HD) patients. However, compared to mineral marker management, there is limited research on bone health based on clinical guidelines [1]. Limited research on the efficacy and safety of bisphosphonates in severe renal impairment restricts osteoporosis treatment options for HD patients [2]. This limitation has delayed focus on the diagnosis and treatment of osteoporosis in this population.

Denosumab is a receptor activator of nuclear factor kappa-Β ligand (RANKL) monoclonal antibody that has been increasingly used in patients with advanced CKD and HD patients due to its independence from renal function [3]. Some studies have focused on short-term improvements in BMD, often noting hypocalcemia [4]. A gap exists in understanding the holistic impact of denosumab on mineral parameters, bone health, and vascular calcification during treatment for CKD-MBD. Denosumab is used for impaired kidney function [5], leading HD patients to start osteoporosis treatment without considering the impact of CKD-MBD, and often outside of dialysis clinics [6]. Therefore, nephrologists require comprehensive information and strategies regarding these treatment approaches and their ramifications.

Our study comprehensively assessed the impact of denosumab on BMD, vascular calcification, and HRQL over a two-year period, alongside specific evaluations of mineral parameters and BTMs at intervals. Limited research exists on the effects of denosumab in osteoporotic patients, necessitating a thorough investigation into its influence on mineral parameters, BTMs, and vascular calcification.

## 2. Materials and Methods

### 2.1. Study Design and Setting

In a prospective observational study at a single center in Korea, we investigated the relationships among denosumab treatment, BMD, biochemical parameters, BTMs, HRQL, and vascular calcification. This study received approval from the Institutional Review Board of Gachon University Gil Medical Center (GBIRB2020–342) and adhered to the Declaration of Helsinki, with written informed consent from all participants.

### 2.2. Study Population

Between March 2020 and February 2022, 185 HD patients at Gachon University Gil Medical Center underwent BMD analysis using dual-energy X-ray absorptiometry (DEXA). Osteoporosis was determined based on Korean Society for Bone and Mineral Research criteria [7] and World Health Organization T-scores for Asian countries, with a T-score ≤ –2.5 in the lumbar spine (LS), femoral neck (FN), or total hip (TH). Patients eligible for denosumab had been undergoing HD for ≥6 months and were free of conditions that would affect their biochemical parameters (e.g., catheters, malignancies, liver disease, infection, parathyroidectomy, or pretreatment with other anti-osteoporotic agents in the preceding 6 months). Patients with recent cardiovascular events, dental procedures, or upcoming dental interventions within 6 months were excluded. In the end, 30 patients were administered a subcutaneous dose of 60 mg/mL denosumab (Prolia^®^ Pre-filled Syringe, Amgen, Inc., Thousand Oaks, CA, USA) every 6 months. Following previous reports [8,9], patients presenting with hypocalcemia (corrected calcium < 8.4 mg/dL) before the denosumab injection received a combination of calcium and cholecalciferol supplements beginning 2 weeks before the administration of denosumab until the tracked corrected calcium level was >10 mg/dL. The corrected calcium level was calculated using Payne’s formula [10]. The other mineral parameters followed the CKD-MBD guidelines [1]. In the end, of the 30 participants, 1 received only two doses over 1 year, while the remaining 29 received the scheduled four doses of denosumab over 2 years. Comparative analyses were conducted with the non-denosumab-administered group as the control, which refers to patients who have maintained stable HD for more than 6 months and whose BMD results do not correspond to osteoporosis or meet the exclusion criteria for denosumab administration.

### 2.3. Fracture Risk Assessment

The 10-year risk of major osteoporotic fracture (MOF) and hip fracture (HF) was computed using the Korean version of the FRAX^®^ calculator, accessible online at http://www.shef.ac.uk/FRAX/tool.aspx?country=25 (accessed on 26 October 2020), based on the completed questionnaires and medical chart review.

### 2.4. Clinical and Laboratory Parameters

Participant data, including demographics, clinical details, comorbidities, and medications, were collected at enrollment by the study coordinator. BMD measurements using DEXA scan were scheduled at baseline and annually for 2 years. Serum lab tests, covering albumin, calcium, phosphate, parathyroid hormone (PTH), bone-specific alkaline phosphatase (BAP), total procollagen-type 1 N-terminal propeptide (tP1NP), osteocalcin, tartrate-resistant acid phosphatase 5b (TRACP5b), C-telopeptide of collagen type 1 (CTx), and T50, were obtained on day 0, week 2, and months 1, 3, 6, 12, and 24. BAP levels were measured using a chemiluminescence immunoassay (Beckman Coulter Inc., Brea, CA, USA), while tP1NP, osteocalcin, TRACP5b, and CTx levels were measured using an electrochemiluminescence immunoassay (Roche Diagnostics, Indianapolis, IN, USA). TRACP5b levels were measured with an enzyme-linked immunosorbent assay (Immunodiagnostics Systems Ltd., Boldon, UK).

### 2.5. Determination of Serum Calcification Propensity (T50)

The T50 value was assessed utilizing a nephelometer (Nephelostar, BMG Labtech, Offenburg, Germany), measuring the time for the transformation from primary to secondary calciprotein particle (CPP) [11,12]. In brief, serum (80 μL) was mixed with NaCl solution (20 μL) and then exposed to supersaturated concentrations of calcium (50 μL) and phosphate (50 μL) solutions in triplicate in a 96-well plate. The Nephelostar, operated via MARS software V2.41, followed the manufacturer’s guidelines. Non-linear regression curves were computed to ascertain the T50 values.

### 2.6. Vascular Calcification Score

Plain X-ray images of the lateral lumbar spine were used to semiquantitatively calculate abdominal aortic calcification (AAC) scores for all subjects [13]. Additionally, denosumab-treated patients underwent electron beam computed tomography (EBCT) to calculate the coronary artery calcium (CAC) score using the Agatston method [14].

### 2.7. Health-Related Quality of Life

The Kidney Disease Quality of Life Instrument-Short Form assesses HRQL in kidney disease patients using the validated Korean version [15]. It includes a disease-specific section for dialysis patients, the Kidney Disease Component Summary (KDCS), which comprises health-related aspects divided into the Physical Component Summary (PCS) and the Mental Component Summary (MCS).

### 2.8. Definition of Outcomes

Primary outcomes were changes in BMD and BTMs following denosumab administration; secondary ones were changes in mineral parameters after denosumab administration in HD patients and the effect on established CKD-MBD treatment strategies that adhere to existing clinical guidelines.

### 2.9. Statistical Analyses

Continuous variables were assessed for normality using the Shapiro–Wilk test. Non-normally distributed variables underwent either log transformation or non-parametric analysis. Normally distributed values are presented as mean ± standard deviation, while non-normally distributed values are reported as median and interquartile range. Group comparisons employed the χ2 test, Student’s *t*-test, or analysis of variance (ANOVA) as appropriate. Within-group changes were evaluated using the paired *t*-test or Wilcoxon’s signed-rank test. All statistical analyses were performed using R software, version 4.3.2., with packages available on the Comprehensive R Archive Network (http://cran.r-project.org, accessed on 31 October 2023). A *p*-value < 0.05 was considered statistically significant.

## 3. Results

### 3.1. Characteristics of the Study Population

Table 1 shows participant characteristics according to denosumab use. Of the 185 subjects, 97 (52.4%) were male, their mean age was 61 ± 12 years, and the average HD duration was 108 months. Comorbidities were prevalent: diabetes mellitus (47.0%), hypertension (58.4%), and previous cardiovascular disease (CVD) (41.1%). The prevalence of osteoporosis was 38.4% in eligible patients (71/185). The denosumab-treated group had mostly female (76.7%) participants. Denosumab use was 7.2% in males and 26.1% in females. Higher single-pool Kt/V (spKt/V) (1.7 vs. 1.6, *p* = 0.004) and lower BMD were observed at the LS, FN, and TH sites (*p* < 0.001). The FRAX^®^ scores for the MOFs (7.6 vs. 12.7, *p* = 0.006) and HFs (3.1 vs. 6.9, *p* = 0.014) were lower. No significant differences in previous fractures, new fractures, comorbidities, medications, or baseline minerals were observed.

BMD was inversely correlated with age and spKt/V and positively correlated with albumin in non-users of denosumab. LS BMD was inversely correlated with ALP, FN, and TH. BMD was inversely correlated with AAC score (Table 2).

### 3.2. Changes in BMD after Denosumab Administration

**Figure 1:**
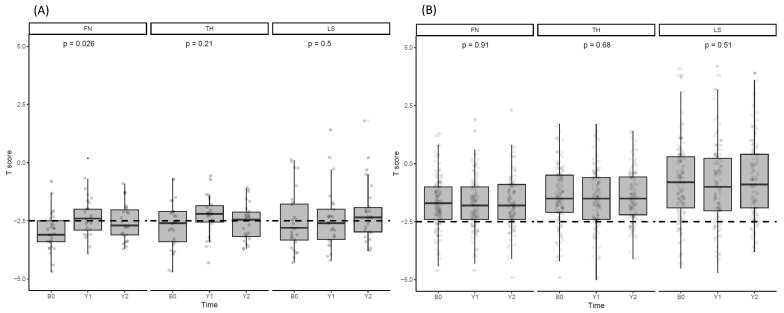
T scores at BMD measurement sites. (**A**) Comparison of BMD T-scores in FN, TH, and LS areas in the denosumab-treated patient group. (**B**) Comparison of BMD T-scores in FN, TH, and LS areas in the untreated group. ANOVA was employed to determine the *p*-values. * *p* ≤ 0.05.

Figure 1 depicts the changes in BMD for LS, FN, and TH before, 1 year after, and 2 years after denosumab treatment. Following 1 year of treatment, BMD increased in FN (−2.97 ± 0.84 vs. –2.38 ± 0.78, *p* = 0.049), which was not sustained during the second year (−2.97 ± 0.84 vs. –2.64 ± 0.94, *p* = 0.285; Figure 1). The treated patients did not exhibit significant changes in BMD at other sites. Similarly, no changes in BMD were observed at any site over the 2 years in patients not taking denosumab (baseline and first year at FN; –1.68 ± 1.13 vs. –1.75 ± 1.16, *p* = 0.662, baseline and second year at FN; –1.68 ± 1.13 vs. –1.67 ± 1.10, *p* = 0.956).

### 3.3. Changes in Bone Turnover Markers after Denosumab Administration

Baseline BTMs showed a positive correlation with serum calcium (TRACP5b: r = 0.607, *p* < 0.001; CTx: r = 0.443, *p* = 0.016; tP1NP: r = 0.520, *p* = 0.004), PTH (TRACP5b: r = 0.582, *p* < 0.001; CTx: r = 0.493, *p* = 0.007; bsALP: r = 0.384, *p* = 0.040; tP1NP: r = 0.745, *p* < 0.001), and alkaline phosphate level (CTx: r = 0.458, *p* = 0.012; bsALP: r = 0.785, *p* < 0.001; tP1NP: r = 0.397, *p* = 0.033) in the treatment group. BMD was not correlated with any of the BTMs (Table 3).

**Figure 2:**
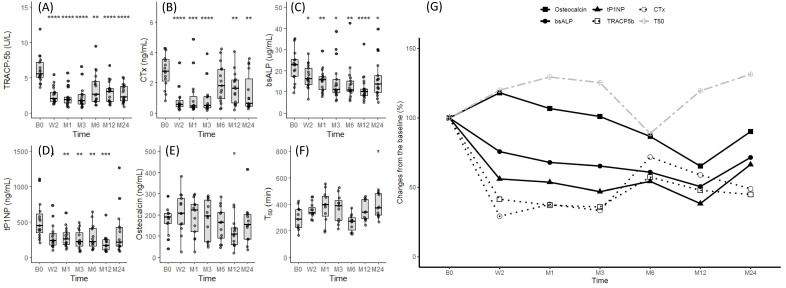
Changes in BTMs and T50 after denosumab administration. Changes in (**A**) TRACP5b, (**B**) CTx, (**C**) BAP, (**D**) tP1NP, (**E**) osteocalcin, and (**F**) T50 observed at baseline, 2 weeks, and 1, 3, 6, 12, and 24 months after denosumab administration. (**G**) Expression of BTMs and T50 as the percentage change from baseline. * *p* ≤ 0.05, ** *p* ≤ 0.01, *** *p* ≤ 0.001, **** *p* ≤ 0.0001.

We compared the changes in bone resorption markers (CTx and TRACP5b) and bone formation markers (osteocalcin, tP1NP, and BAP) in treated patients relative to baseline (Figure 2). Both decreased by <50% 2 weeks after denosumab was initiated, and this was maintained for about 3 months; it then increased near the next dosing at 6 months. Among the bone formation markers, tP1NP and BAP gradually decreased during treatment compared to the more rapid changes in the bone resorption markers. Osteocalcin initially increased and then gradually decreased with the other markers.

### 3.4. Changes in Mineral Parameters after Denosumab Administration

**Figure 3:**
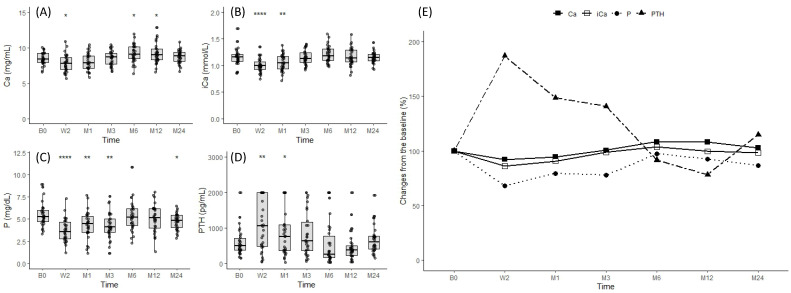
Changes in mineral parameters after denosumab administration. Changes in (**A**) calcium, (**B**) ionized calcium, (**C**) phosphate, and (**D**) PTH were observed at baseline, 2 weeks, and 1, 3, 6, and 12 months after denosumab administration. (**E**) Expression of mineral parameters as a percentage change from baseline. * *p* ≤ 0.05, ** *p* ≤ 0.01, **** *p* ≤ 0.0001.

Figure 3 depicts the changes in serum levels of calcium, ionized calcium, phosphate, and PTH after denosumab was administered. A corrected calcium <8.4 mg/dL triggered calcium and cholecalciferol supplementation. Calcium (8.49 ± 0.89 mg/dL vs. 7.81 ± 1.23 mg/dL, *p* = 0.030; Figure 3A) and ionized calcium levels (1.17 ± 0.16 mg/dL vs. 1.00 ± 0.13 mg/dL, *p* < 0.001; Figure 3B) decreased by the second week after the first injection but normalized within the first month. Phosphorus levels decreased rapidly after treatment, sustaining a maximum drop for 2 weeks (5.48 ± 1.33 mg/dL vs. 3.71 ± 1.31 mg/dL, *p* < 0.001; Figure 3C). This effect persisted for 3 months but was normalized by the next denosumab dosing at 6 months (5.48 ± 1.33 mg/dL vs. 4.25 ± 1.47 mg/dL, *p* = 0.374; Figure 3C). PTH surged immediately after the treatment and peaked at 2 weeks (587.51 ± 388.91 pg/mL vs. 1099.41 ± 727.78 pg/mL, *p* < 0.001; Figure 3D). Despite active treatments, such as vitamin D and cinacalcet, following the CKD-MBD clinical guidelines, this increase persisted for the first months (587.51 ± 388.91 pg/mL vs. 874.03 ± 659.98 pg/mL, *p* = 0.048; Figure 3D). Levels tended to decline after 3 months to near baseline (587.51 ± 388.91 pg/mL vs. 874.03 ± 659.98 pg/mL, *p* = 0.048; Figure 3D). Change rates are illustrated in Figure 3E.

**Figure 4:**
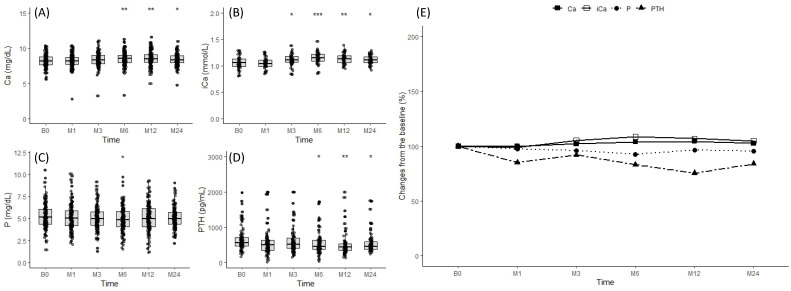
Changes in mineral parameters in the untreated group. Changes in (**A**) calcium, (**B**) ionized calcium, (**C**) phosphate, and (**D**) PTH observed at baseline, 2 weeks, and 1, 3, 6, and 12 months. (**E**) Expression of mineral parameters as a percentage change from baseline. * *p* ≤ 0.05, ** *p* ≤ 0.01, *** *p* ≤ 0.001.

HD patients following the CKD-MBD guidelines who were not taking denosumab exhibited no significant fluctuations in mineral parameters (Figure 4A–E).

### 3.5. Relationship between T50, Mineral Parameters, and Bone Turnover Markers after Denosumab Administration

The decreases in bone metabolism markers and calcium and phosphate levels were associated with an increase in T50. Subsequently, T50 tended to decrease as the next dosing cycle approached, and the bone metabolism markers and mineral parameters normalized toward baseline levels (Figure 2F,G).

### 3.6. Effect of Denosumab on Vascular Calcification

**Figure 5:**
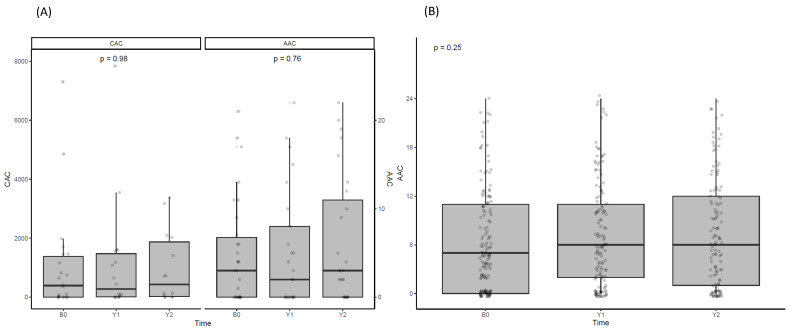
Changes in vascular calcification scores after denosumab administration. (**A**) Changes in CAC measured using EBCT and AAC measured using plain X-ray in the denosumab-treated group at baseline, 12, and 24 months. (**B**) Representation of changes in AAC measured using plain X-ray in the untreated group at the same observation points. ANOVA was employed to represent the *p*-values.

Vascular calcification was measured in the treated group via EBCT and plain X-ray, while only plain X-ray was conducted in the untreated group (Figure 5). No significant changes in vascular calcification were observed in either group during the observation period.

### 3.7. Effect of Denosumab on Quality of Life

**Figure 6:**
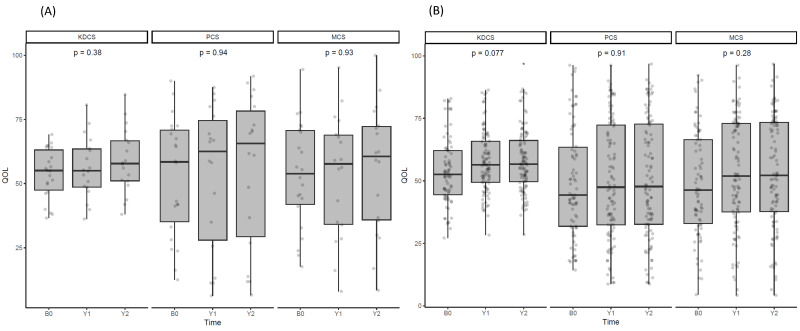
Changes in HRQL after denosumab administration. (**A**) Scores on the KDCS, PCS, and MCS items for HRQL in the denosumab-treated group and changes at baseline, 12, and 24 months. (**B**) Representation of the changes in HRQL measured at the same observation points in the untreated group. ANOVA was employed to represent the *p*-values. * *p* ≤ 0.05.

HRQL scores were not significantly different between the groups at baseline (Figure 6). When assessed annually over the 2 years, neither group exhibited a significant difference in the KDCS, PCD, or MCS categories.

## 4. Discussion

The prevalence of osteoporosis in HD patients was 38.4% (71 of 185), with 16.2% receiving denosumab (7.2% of males and 26.1% of females). Denosumab boosted FN BMD after 12 months, which faded after 2 years. BTMs decreased after denosumab was administered; resorption markers initially dropped and then increased, and formation markers steadily decreased. T50 increased as BTMs decreased. Hypocalcemia (<8.4 g/dL) post-denosumab was prevented by 2 weeks of supplementation. PTH initially surged and improved despite vitamin D and calcimimetic administration. Vascular calcification scores and HRQL did not significantly change in either group.

ESKD patients often face bone disorders, leading to complications like fractures [16], attributable to irregular bone turnover and diminished BMD. While bone biopsy is the gold standard for turnover diagnosis, its invasiveness hampers practicality [1]. Specific hormones like PTH and fibroblast growth factor-23 add complexity to bone marker regulation in ESKD [17], posing challenges in diagnosing and treating various CKD-MBD factors associated with BMD reduction.

Our study determined denosumab treatment targets based solely on BMD. However, additional assessment tools are necessary to evaluate fracture risk. A 10-year intervention threshold of 20% for MOF and 3% for HF is employed when selecting patients for osteoporosis treatment using the FRAX^®^ tool. We made this selection because FRAX^®^ exhibits superior performance in identifying patients unlikely to experience major osteoporotic or hip fractures within the next 10 years [18]. However, this discriminatory method has only been applied to a subset of HD patients in studies with short observation periods [19]. Therefore, further research is needed to assess its value as a tool for discerning indications for osteoporosis treatment and preventing fracture.

Osteoporosis has received less attention in ESKD patients due to the limitations of bisphosphonate use [20]. The development of denosumab has led to increased efforts in diagnosing osteoporosis in ESKD patients [21,22], with previous reports indicating an associated increase in BMD [3,4,9,23]. Our study demonstrated improved BMD in the FN area 1 year after denosumab was administered. However, the second-year results indicated a trend of non-sustainability. Long-term follow-up studies post denosumab administration have revealed that the initial improvements in hip region BMD gradually diminish, and discontinuing the drug over the following year leads to a decline in BMD [23]. These findings suggest the need for prolonged administration to maintain improved BMD status. The absence of strategies to prevent the decrease in BMD after discontinuing denosumab underscores the need for further research in this area.

Previous research has consistently indicated a decrease in BTMs following denosumab administration [3,9,24]. This decline persists until approximately 3 months post-administration, after which the BTMs stabilize as the next dosing at 6 months approaches [25]. Given that denosumab inhibits bone resorption by targeting RANKL, our study reflected this, showing a swift reduction in bone resorption markers compared to bone formation markers in the early stages of denosumab treatment. This led to a secondary reduction in bone formation markers, which was repeated after each administration.

T50, reflecting the transition time from primary to secondary CPPs in vitro [26], is a proposed serum marker indicating the propensity for calcification. A shorter T50 suggests a higher tendency for calcification, linked to increased cardiovascular disease (CVD) risk and all-cause mortality in CKD patients [11]. A prior cross-sectional study indicated a weak correlation between T50 and BMD [12]. The increase in T50 observed with denosumab treatment suggests prolonged maturation of CPP due to reduced bone resorption and lower calcium and phosphate levels. Early changes in bone metabolism post denosumab administration may affect the T50 level. Given the limited data on these effects on vascular health, further research is crucial for a comprehensive understanding of this area.

Denosumab often leads to hypocalcemia because of inhibited RANKL [2]. Similar to previous research [3,8,9,25], we noted an early sharp drop in calcium levels after denosumab was initiated. Our treatment approach of providing calcium and cholecalciferol until the corrected calcium of 10 mg/dL is reached effectively regulated hypocalcemia. Despite efforts to mitigate hypocalcemia [3,17], the significant increase in PTH post-denosumab administration in our study suggests that mechanisms beyond hypocalcemia triggered this increase. As a mechanism to explain this, the possibility of an osteoanabolic effect of PTH due to a decrease in cortical porosity in the early stages of denosumab administration has been suggested [27,28], but the exact mechanism is not yet known.

CKD-MBD, coupled with osteoporosis and a high RANKL level, heightens bone loss, vascular inflammation, and calcification risk. Administering denosumab to CKD-MBD patients with osteoporosis lowered calcium and phosphate levels and inhibited vascular inflammation and calcification. In a small-scale study involving 21 HD patients, no improvement in CAC score was detected 6 months after denosumab administration when measured using EBCT [29].

The authors noted an inverse correlation between serum alkaline phosphatase level and coronary artery calcium (CAC) score, suggesting that the selective cessation of osteoclasts, along with intact osteoblast function and a high bone turnover rate, might create a reverse calcium paradox. This could lead to regressed ectopic calcification, stabilized vascular calcification, and increased bone mass [30,31]. However, our 2-year study found no significant changes in CAC. In the non-denosumab group, there was a negative correlation between AAC and BMD_FN as well as BMD_TH. Surprisingly, in the denosumab group, no significant correlation was observed. The absence of a negative correlation in the denosumab treatment group may be attributed to their relatively lower bone mineral density. Therefore, this complexity implies mechanisms beyond straightforward changes in calcium content, underscoring the need for further investigation.

Enhancing HRQL and preventing fractures in HD patients with multiple comorbidities is vital when treating CKD-MBD, including osteoporosis. In a recent 24-month study with 332 non-dialysis osteoporotic patients, denosumab was associated with improved BMD and HRQL [32]. While the BMD-HRQL link is promising, the lack of a control group and different patient populations necessitate further research to understand the factors involved in improving HRQL post-denosumab administration, particularly in patients undergoing HD. In our study, denosumab slightly improved FN BMD but did not significantly affect HRQL. The effects of comorbidities and other factors remain to be explored.

This first-of-its-kind Korean study examined denosumab’s impact on BMD, mineral parameters, vascular calcification, and HRQL in HD patients, shedding light on osteoporosis treatment. However, this study has limitations, including a relatively small denosumab group, emphasis on measurable changes like BMD, and a potentially short 24-month study period to assess long-term denosumab safety. Additionally, there is a possibility of bias due to the selection of a control group that may not be well-matched to the denosumab treatment group. Furthermore, the lack of analysis of critical events such as new fractures or mortality after denosumab is a notable gap, underscoring the necessity for comprehensive exploration in future studies.

## 5. Conclusions

The initial effect of denosumab on BMD was less pronounced in HD patients. Denosumab affected BTMs by managing calcium and phosphate levels effectively, consistent with the CKD-MBD strategy. Notably, PTH levels initially increased. HD patients without denosumab maintained stable control over calcium, phosphate, and PTH levels for 24 months, but there were no significant improvements in vascular calcification or HRQL. Larger studies are necessary to appraise the changes in BMD and reduction in fracture risk associated with denosumab in osteoporotic HD patients.

## Figures and Tables

**Table 1:** Baseline characteristics of the study group.

	Total (*n* = 185)	Control (*n* = 155)	Denosumab (*n* = 30)	*p*-Value
Age, yr	61.0 ± 12.3	60.9 ± 12.1	62.1 ± 13.3	0.621
Male, *n* (%)	97 (52.4)	90 (58.1)	7 (23.3)	0.001
HD duration (month)	108 (64–139)	106 (64–136)	119 (71–143)	0.379
BMI, kg/cm^2^	23.6 ± 3.8	23.8 ± 3.9	22.3 ± 3.3	0.046
Smoking, *n* (%)	29 (15.6)	28 (18.0)	1 (3.3)	0.109
DM, *n* (%)	87 (47.0)	75 (48.4)	12 (40.0)	0.520
HTN, *n* (%)	108 (58.4)	91 (58.7)	17 (56.7)	0.996
CVD, *n* (%)	76 (41.1)	64 (41.3)	12 (40.0)	1.000
spKt/V	1.6 [1.4–1.8]	1.6 [1.4–1.8]	1.7 [1.5–2.0]	0.004
RAS blockade, *n* (%)	79 (42.7)	69 (44.5)	10 (33.3)	0.351
CCB, *n* (%)	81 (43.8)	70 (45.2)	11 (36.7)	0.511
β-blocker, *n* (%)	80 (43.2)	71 (45.8)	9 (30.0)	0.162
P binder	132 (71.4)	110 (71.0)	22 (73.3)	0.967
Statin	71 (38.4)	61 (39.4)	10 (33.3)	0.678
Vitamin D analogues	123 (66.5)	101 (65.2)	22 (73.3)	0.511
Cinacalcet	17 (9.1)	14 (9.0)	3 (10.0)	0.873
Hb, g/dL	10.8 ± 1.3	10.9 ± 1.3	10.6 ± 1.1	0.221
Alb, g/dL	4.0 ± 0.3	4.0 ± 0.4	4.0 ± 0.3	0.696
Cholesterol, mg/dL	136.7 ± 34.8	136.2 ± 36.1	139.4 ± 27.6	0.645
TG, mg/dL	102.1 ± 72.6	104.2 ± 75.0	91.1 ± 58.1	0.367
hsCRP, mg/dL	0.2 [0.1–0.4]	0.2 [0.1–0.4]	0.1 [0.0–0.3]	0.447
Ca, mg/dL	8.2 ± 0.9	8.2 ± 0.9	8.5 ± 0.9	0.123
P, mg/dL	5.3 ± 1.4	5.2 ± 1.4	5.5 ± 1.3	0.408
VD_25_, ng/mL	17.2 ± 9.8	17.5 ± 9.2	15.8 ± 12.3	0.490
VD_1,25_, pg/mL	5.9 ± 7.1	6.0 ± 7.2	5.6 ± 6.8	0.789
PTH, pg/mL	564.8 ± 380.0	560.4 ± 379.3	587.5 ± 388.9	0.721
ALP, U/L	108.3 ± 64.5	108.2 ± 66.9	109.3 ± 50.9	0.927
T_50_, min	296.3 ± 85.3	298.6 ± 86.5	284.8 ± 79.3	0.419
BMD_LS, T score	–1.1 ± 1.7	–0.8 ± 1.7	–2.6 ± 1.3	<0.001
BMD_FN, T score	–1.9 ± 1.2	–1.7 ± 1.1	–2.9 ± 0.9	<0.001
BMD_TH, T score	–1.6 ± 1.3	–1.4 ± 1.3	–2.7 ± 1.0	<0.001
FRAX_MOF	8.5 ± 6.3	7.6 ± 5.2	12.7 ± 9.0	0.006
FRAX_HF	3.8 ± 4.6	3.1± 3.4	6.9 ± 7.3	0.014
Previous FX_Hx	23 (12.4)	17 (11.0)	6 (20.0)	0.285
FX_Event	7 (3.8)	5 (3.3)	2 (6.7)	0.703
AAC	5.0 [0.0–11.0]	5.0 [0.0–11.0]	3.0 [0.0–9.0]	0.248
KDCS	53.7 ± 12.6	53.9 ± 13.3	52.8 ± 9.5	0.725
PCD	49.8 ± 22.3	49.4 ± 51.2	51.2 ± 23.6	0.753
MCS	49.4 ± 21.0	48.7 ± 21.0	52.2 ± 21.2	0.507

HD, hemodialysis; BMI, body mass index; DM, diabetes; HTN, hypertension; CVD, cardiovascular disease; spKtV, single-pool Kt/V; RAS, renin–angiotensin–aldosterone; CCB, calcium channel blocker; P binder, phosphate binder; Hb, hemoglobin; Alb, albumin; TG, triglyceride; hsCRP, highly selective C-reactive protein; Ca, calcium; P, phosphate; VD_25_, 25-hydroxyvitamin D; VD_1,25_, 1,25-dihydroxyvitamin D; PTH, parathyroid hormone; ALP, alkaline phosphatase; BMD_LS, bone mineral density of the lumbar spine; BMD_FN, bone mineral density of the femoral neck; BMD_TH, bone mineral density of the total hip; FRAX_MOF, fracture risk assessment for 10-year risk of major osteoporotic fracture; FRAX_HF, fracture risk assessment for 10-year risk of hip fracture; Previous FX_Hx, previous fracture history; FX_Event; new development of fracture event during observation period; AAC, abdominal aortic calcification; KDCS, kidney disease component summary; PCD, physical component summary; MCS, mental component summary.

**Table 2:** Correlations between BMD and other variables.

	Age	spKtV	Alb	hsCRP ^a^	Ca	P	PTH	ALP	BMD_LS	BMD_FN	BMD_TH	AAC	T_50_
Age	1.000												
spKtV	0.196 *	1.000											
Alb	–0.229 *	0.001	1.000										
hsCRP ^a^	0.043	–0.121	–0.159	1.000									
Ca	0.002	–0.010	0.291 *	–0.126	1.000								
P	–0.340 *	–0.206 *	0.243 *	0.085	0.058	1.000							
PTH	–0.218 *	–0.196 *	0.136	–0.047	0.161	0.321 *	1.000						
ALP	0.044	–0.051	0.078	0.028	0.064	–0.009	0.341 *	1.000					
BMD_LS	–0.211 *	–0.215 *	0.085	0.031	–0.006	0.176 *	–0.094	–0.258 *	1.000				
BMD_FN	–0.523 *	–0.364 *	0.189 *	0.025	0.077	0.158	0.041	0.040	0.590 *	1.000			
BMD_TH	–0.434 *	–0.427 *	0.212 *	0.048	0.051	0.1531	0.012	–0.091	0.632 *	0.907 *	1.000		
AAC ^a^	0.433 *	0.002	–0.098	0.080	0.097	–0.038	–0.050	0.122	–0.012	–0.311 *	–0.291 *	1	
T_50_	–0.008	–0.025	0.316 *	–0.207 *	0.016	–0.143	0.002	–0.162	0.092	0.011	0.133	–0.024	1

^a^ Data for hsCRP and AAC were log-transformed. * *p* < 0.05. Alb, albumin; hsCRP, highly selective C-reactive protein; Ca, calcium; P, phosphate; PTH, parathyroid hormone; ALP, alkaline phosphatase; BMD_LS, bone mineral density—lumbar spine; BMD_FN, bone mineral density—femur neck; BMD_TH, bone mineral density—total hip; AAC, abdominal aortic calcification.

**Table 3:** Correlations between BTMs and other variables in patients with denosumab administration.

	Ca	P	PTH	ALP	BMD_LS	BMD_FN	BMD_TH	AAC	T_50_	TRACP5b	CTx	BAP	tP1NP	OC
Ca	1.000													
P	0.032	1.000												
PTH	0.335	0.431 *	1.000											
ALP	0.221	–0.247	0.339	1.000										
BMD_LS	0.216	0.015	0.128	–0.055	1.000									
BMD_FN	0.055	0.249	0.081	–0.429 *	0.016	1.000								
BMD_TH	–0.075	0.257	0.065	–0.429 *	0.211	0.837 *	1.000							
AAC ^a^	–0.089	–0.059	–0.174	0.168	0.077	–0.364	–0.167	1.000						
T_50_	–0.052	–0.124	–0.469 *	–0.062	0.083	–0.098	–0.008	0.496 *	1.000					
TRACP5b	0.607 *	0.183	0.582 *	0.366	0.142	0.063	0.021	–0.137	–0.102	1.000				
CTx	0.443 *	–0.018	0.493 *	0.458 *	–0.081	0.049	–0.034	–0.126	–0.307	0.576 *	1.000			
BAP	0.258	–0.232	0.384 *	0.785 *	–0.069	–0.205	–0.274	0.055	0.011	0.553 *	0.580 *	1.000		
tP1NP	0.520 *	0.369 *	0.745 *	0.397 *	0.158	−0.064	−0.047	0.060	–0.253	0.720 *	0.625 *	0.543 *	1.000	
OC	0.200	–0.103	–0.191	0.183	0.240	−0.005	0.183	0.333	0.410 *	0.013	–0.119	0.103	–0.050	1.000

^a^ Data for hsCRP and AAC were log-transformed. * *p* < 0.05. hsCRP, highly selective C-reactive protein; Ca, calcium; P, phosphate; PTH, parathyroid hormone; ALP, alkaline phosphatase; BMD_LS, bone mineral density—lumbar spine; BMD_FN, bone mineral density—femur neck; BMD_TH, bone mineral density—total hip; AAC, abdominal aortic calcification; TRACP5b, tartrate-resistant acid phosphatase 5b (TRACP5b); CTx, C-telopeptide of collagen type 1; BAP, bone-specific alkaline phosphatase; tP1NP, total procollagen-type 1 N-terminal propeptide; OC, osteocalcin.

## Data Availability

The data presented in this study are available on request from the corresponding author.
